# Using the Redundant Convolutional Encoder–Decoder to Denoise QRS Complexes in ECG Signals Recorded with an Armband Wearable Device

**DOI:** 10.3390/s20164611

**Published:** 2020-08-17

**Authors:** Natasa Reljin, Jesus Lazaro, Md Billal Hossain, Yeon Sik Noh, Chae Ho Cho, Ki H. Chon

**Affiliations:** 1 Department of Biomedical Engineering, University of Connecticut, Storrs, CT 06269, USA; natasa.reljin@gmail.com (N.R.); jlazarop@unizar.es (J.L.); md.b.hossain@uconn.edu (M.B.H.); ccho.ct@gmail.com (C.H.C.); 2 Biomedical Signal Interpretation and Computational Simulation (BSICoS) Group, Aragon Institute of Engineering Research (I3A), IIS Aragon, University of Zaragoza, 50009 Zaragoza, Spain; 3 CIBER in Bioengineering, Biomaterials and Nanomedicine (CIBER-BBN), 28029 Madrid, Spain; 4 College of Nursing, University of Massachusetts Amherst, Amherst, MA 01003, USA; ynoh@umass.edu; 5 Department of Electrical and Computer Engineering, University of Massachusetts Amherst, Amherst, MA 01002, USA; ynoh@umass.edu

**Keywords:** denoising algorithm, redundant convolutional encoder–decoder (R-CED), wearable devices, electrocardiogram (ECG), motion artifacts, signal-to-noise ratio (SNR), ratio of power, cross-correlation

## Abstract

Long-term electrocardiogram (ECG) recordings while performing normal daily routines are often corrupted with motion artifacts, which in turn, can result in the incorrect calculation of heart rates. Heart rates are important clinical information, as they can be used for analysis of heart-rate variability and detection of cardiac arrhythmias. In this study, we present an algorithm for denoising ECG signals acquired with a wearable armband device. The armband was worn on the upper left arm by one male participant, and we simultaneously recorded three ECG channels for 24 h. We extracted 10-s sequences from armband recordings corrupted with added noise and motion artifacts. Denoising was performed using the redundant convolutional encoder–decoder (R-CED), a fully convolutional network. We measured the performance by detecting R-peaks in clean, noisy, and denoised sequences and by calculating signal quality indices: signal-to-noise ratio (SNR), ratio of power, and cross-correlation with respect to the clean sequences. The percent of correctly detected R-peaks in denoised sequences was higher than in sequences corrupted with either added noise (70–100% vs. 34–97%) or motion artifacts (91.86% vs. 61.16%). There was notable improvement in SNR values after denoising for signals with noise added (7–19 dB), and when sequences were corrupted with motion artifacts (0.39 dB). The ratio of power for noisy sequences was significantly lower when compared to both clean and denoised sequences. Similarly, cross-correlation between noisy and clean sequences was significantly lower than between denoised and clean sequences. Moreover, we tested our denoising algorithm on 60-s sequences extracted from recordings from the Massachusetts Institute of Technology-Beth Israel Hospital (MIT-BIH) arrhythmia database and obtained improvement in SNR values of 7.08 ± 0.25 dB (mean ± standard deviation (sd)). These results from a diverse set of data suggest that the proposed denoising algorithm improves the quality of the signal and can potentially be applied to most ECG measurement devices.

## 1. Introduction

Electrocardiogram (ECG) is a leading tool used for monitoring and diagnosing cardiac diseases. Many heart conditions are intermittent and paroxysmal in nature and can be detected and observed only with continuous long-term ECG monitoring. Patients are typically given a Holter monitor with wet electrodes connected to it via wires for continuous monitoring. This device has several drawbacks: (1) the monitor has to be carried in a pouch by the patient, which makes it inconvenient; (2) the subjects’ body movements can cause concomitant shifting of ECG leads, which may negatively affect electrodes’ contact with the skin, thereby reducing signal fidelity [1]; (3) wet electrodes can cause skin irritation, bacterial growth, and signal degradation with prolonged duration [2]. Another option for continuous monitoring is recently developed patch devices [3]. Even though these devices are less bulky and do not have wires, they still have wet electrodes that are attached to the chest via adhesive, which are known to cause skin irritation with prolonged use.

To overcome the limitations of Holter monitors and patch devices, a wearable armband designed to be worn on the upper left arm has recently been developed in our lab [4]. The armband contains three sets of carbon-based dry electrodes [2] that, when used differentially, can acquire three ECG channels simultaneously. The advantages of this device are no sources of skin irritation and no need for wires. Due to the fact that the armband is positioned over two main limb muscles, the biceps and triceps, ECG signals could theoretically be corrupted with significant motion noise artifacts and muscle noises. However, it was shown that the armband is a suitable device for long-term ECG monitoring, especially during nighttime recording, when the device provides ~95% usable ECG data [4]. For daytime recordings to be usable, an innovative signal processing approach was developed to obtain ~75% of the data [4]. However, due to muscle and movement artifacts, better approaches are needed to utilize more usable data with these types of wearable devices.

Many techniques have been proposed for denoising ECG signals. Guler and Ubeyli used a model based on a neural network for ECG denoising [5]. Several studies used adaptive filtering as a denoising algorithm [6,7], while Tracey and Miller used a nonlocal means approach [8]. Selesnick proposed a sparsity-assisted signal smoothing (SASS) method that combined low-pass filtering and generalized total-variation denoising approaches [9]. He et al. proposed a denoising algorithm that uses independent component analysis (ICA) [10]. Algorithms based on various versions of the Kalman filter have been proposed [11,12,13]. Denoising algorithms based on wavelet transform are well explored in the literature [14,15,16,17,18], while some researchers proposed denoising algorithms based on empirical mode decomposition (EMD) [19,20,21,22]. Akhbari et al. proposed a method based on a nonlinear dynamic model, which uses Gaussian functions [23]. Zhou et al. proposed an algorithm that approximates an ECG signal as the linear combination of structures and removes the additive random noise and baseline wandering [24]. Various variations of denoising autoencoders were used in [25,26].

Presence of noise in ECG signals is unavoidable, and it is difficult and a challenging task to separate it from the signal. Having in mind that noise has a complex structure and often consists of various components that have different, usually unknown frequency distributions, common and simple filtering techniques (such as linear filtering) typically do not provide effective results. To this end, we applied a denoising algorithm based on the redundant convolutional encoder–decoder (R-CED) network [27]. In order to show the robustness of our algorithm, we applied it to: (1) 24-h ECG recordings acquired with a wearable armband device developed in our lab when different noise types of varying levels were added to the data segments that were designated as clean; (2) 24-h ECG recordings corrupted with motion artifacts that are acquired with a wearable armband device developed in our lab; (3) recordings from the Massachusetts Institute of Technology-Beth Israel Hospital (MIT-BIH) arrhythmia database [28,29]. By doing so, we show that our algorithm was able to remove various noise types (deliberately added colored noises, and motion artifacts), as they simulate various scenarios that can occur in ECG signals acquired with different devices.

The evaluation of the denoising algorithm was performed by R-peak detection in clean, noisy, and denoised sequences and by calculating signal quality indices: signal-to-noise ratio (SNR) improvement, the ratio of power, and cross-correlation with respect to the clean sequences. While we used the armband for the development of the denoising algorithm, the method can potentially be applied to the data from any other ECG device including Holter and patch monitors. We highlight the use of an armband in this application because it provides a good test for the denoising algorithm, since the device is not common and more prone to noise, hence, it is a good testbed. Our rationale is that if the denoising algorithm works for the armband, it should be equally or more applicable to commonly available ECG devices. While the existing denoising methods require a signal from an external ECG recording device to denoise the signal, our algorithm uses only the ECG recordings from the armband wearable device, which is one of the main advantages and innovations of our algorithm. Finally, the main purpose of denoising was to obtain reliable QRS complexes, so that heart-rate estimates could be obtained. Heart rates are important clinical information, as they can be used for analysis of heart-rate variability and cardiac arrhythmia detection including atrial fibrillation [30].

## 2. Materials and Methods

### 2.1. Data Acquisition

**Figure 1:**
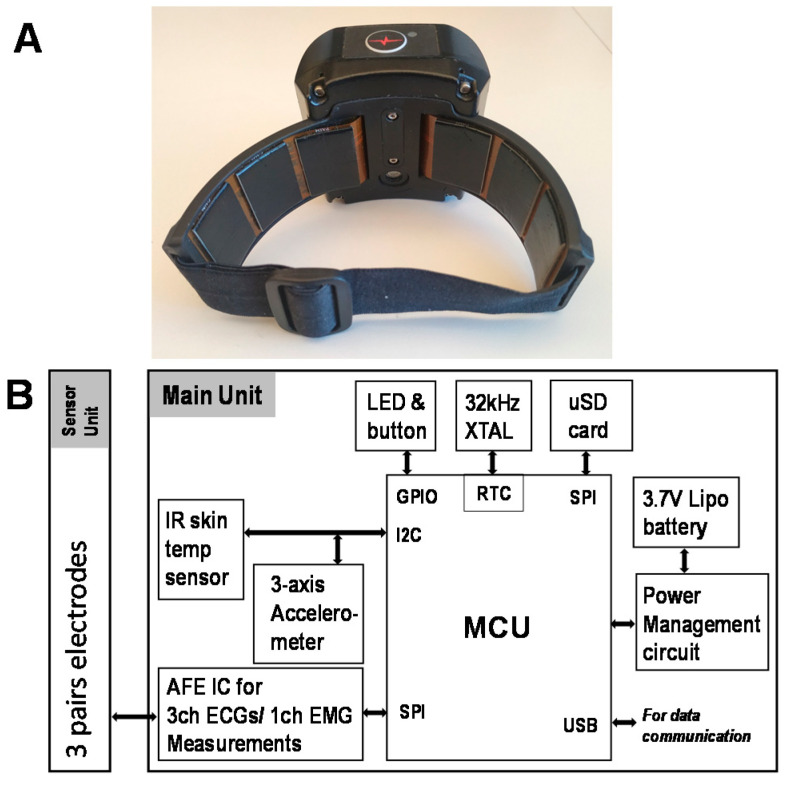
(**A**) Armband. (**B**) Schematic diagram of the armband.

Data were recorded with the wearable armband, which is designed to be worn on the upper left arm [4]. The armband consists of two parts: (1) the main unit, which has a microcontroller (MCU), μSD memory unit, power management, and analog front end (AFE) integrated circuit (IC) unit and (2) a sensor unit, which contains three sets of carbon-black dry electrodes. These electrodes are used for recording three ECG channels and one electromyogram (EMG) channel simultaneously, and these data are stored on the μSD card. Figure 1 shows the armband and its schematic diagram.

All signals were continuously recorded for 24 h from one male subject, while performing a normal daily routine, with a sampling frequency *f_s_* = 1000 Hz. In addition to the armband device, reference ECG signals were simultaneously recorded with a widely available Holter monitor (Rozinn RZ 153+, Glendale, NY, USA). Note that the recordings from Holter monitor were used only to confirm that recordings obtained by armband device do indeed correspond to the former recordings and have ECG morphology. All human study protocols were approved by the University of Connecticut Institutional Review Board (H16-107).

**Figure 2:**
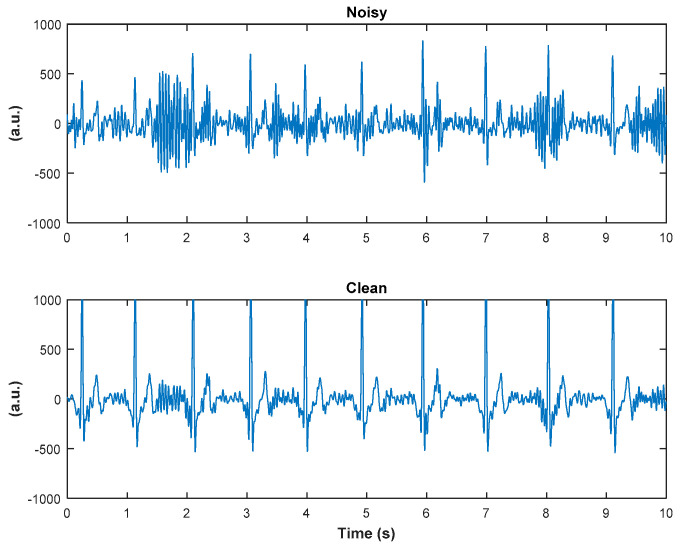
An illustrative example of a 10-s sequence from a noisy channel, denoted as noisy (top panel) and the corresponding sequence from a clean channel, denoted as clean (bottom panel), of armband data.

The ECG signals were first down-sampled to 256 Hz and, then, filtered with a band-pass filter with 3 Hz and 25 Hz as the cut-off frequencies in order to remove most of the muscle-induced artifacts from the signals. The positions of the electrodes in the armband and their placement on the arm (over biceps and triceps) provided at least one ECG channel with the cleanest signal, and one that was the most corrupted with noise. It is worth mentioning that we did not encounter a situation where all channels yielded clean signals, nor all channels yielded the noise-corrupted signals. This is similar in concept to a Holter monitor, which has several channels of which, due to different electrode locations, a particular channel provides the best quality ECG signal at a given time. In the remainder of this paper, the cleanest ECG channel is denoted as ‘clean,’ and is considered as a reference, while the noise-corrupted channel as ‘noisy.’ Adjudication of clean versus noisy channels was determined visually by three skilled persons (authors N.R., K.H.C., and J.L.). If P, Q, R, S, and T waves could be clearly identified and visible during the majority of recording, then the channel is denoted as clean. If the presence of these waves is not visible, the channel is labeled as noisy. Figure 2 shows an illustrative example of a 10-s sequence from a clean channel and its corresponding sequence from a noisy channel.

### 2.2. Extraction of Armband Sequences for Adding Colored Noises

We wanted to explore how our algorithm would perform if noise was added to clean sequences. To this end, we added colored noises of different levels to sixteen 10-s sequences from the clean channel. Added noises included: white, pink, blue, purple, and brown, with SNR values of −5 dB and −7 dB for white, pink, blue, and purple noises and −15 dB and −17 dB for brown noise. These noise levels were chosen so that they amply buried the QRS complexes of the ECG signal. White noise is common background noise present in any device with limited bandwidth. Pink noise is related to the power line interference, while electrode movement noise and baseline wander relate to brown noise [31,32]. Acquisition systems perform conversion from analog to digital signal, and thus, blue, purple, or both noises are present. As a training set, we used 10 sequences, each corrupted with five noises and both SNR values to form 100 training sequences. The remaining 6 sequences, corrupted with all the noise types (60 sequences in total), were used as a test set.

### 2.3. Extraction of Armband Sequences with Motion Noise Artifacts

We extracted 69 ten-second sequences corrupted with motion artifacts from the noisy channel, and the corresponding 69 ten-second sequences from the clean channel, for a total of 138 sequences. Adjudication of clean versus noisy channels was determined visually by three of the authors (N.R., K.H.C. and J.L.). Furthermore, this dataset was divided into training and test sets. For the training set, we used 60% of the sequences, i.e., 41 noisy and their corresponding 41 clean sequences, for a total of 82 ten-second sequences, while the remaining 28 noisy sequences were used as a test set.

### 2.4. Extraction of Sequences from the MIT-BIH Arrhythmia Database

We also used recordings from the MIT-BIH arrhythmia database [28,29]. Twenty-one 60-s sequences with added white noise of SNR value −5 dB were used, where 11 were used to form a training set, while the remaining 10 formed a test set. Note that the duration of sequences, as well as added noise and SNR value in this Section, were chosen for comparison purposes.

### 2.5. Denoising Algorithm

The redundant convolutional encoder–decoder (R-CED) was used as a denoising algorithm [27]. This type of convolutional neural network contains repetitions of convolutional, batch normalization, and rectified linear unit (ReLU) layers. The last layer in R-CED is a convolutional layer. It was shown in the literature that R-CED achieved better results than recurrent networks even with a smaller number of network parameters when one-dimensional signals were denoised [27], thus making it a suitable algorithm for denoising of ECG signals, which can be embedded in the wearable device. Before training the network, all sequences in the dataset were transformed to the frequency domain by applying the 256-point short-time Fourier Transform (STFT) with a 192-point (75%) Hamming window overlap. We then reduced the size of the STFT magnitude vectors to 129.

During the training stage, the predictor input was formed by merging 8 consecutive STFT magnitude spectra obtained from noisy sequences from the training set, while the target signal contained magnitude spectra of the corresponding clean sequences. Both predictor and target signals were normalized to zero mean and unit variance. We trained the R-CED network with 16 convolutional, batch normalization, and ReLU layers. We used a grid search method for determining parameters for the denoising algorithm to avoid overfitting and to obtain the best results [33]. To this end, we varied the following parameters: the number of epochs, mini-batch size, drop in learning rate after each epoch, and an optimizer. The network was trained using the back-propagation with a stochastic gradient descent with momentum (SGDM) optimizer, a mini-batch size of 128, 12 epochs, and a decreased learning rate by 0.95 every time an epoch has passed. The implementation of the denoised algorithm was performed in MATLAB^®^ (R2019a, The Mathworks, Inc., Natick, MA, USA).

**Figure 3:**
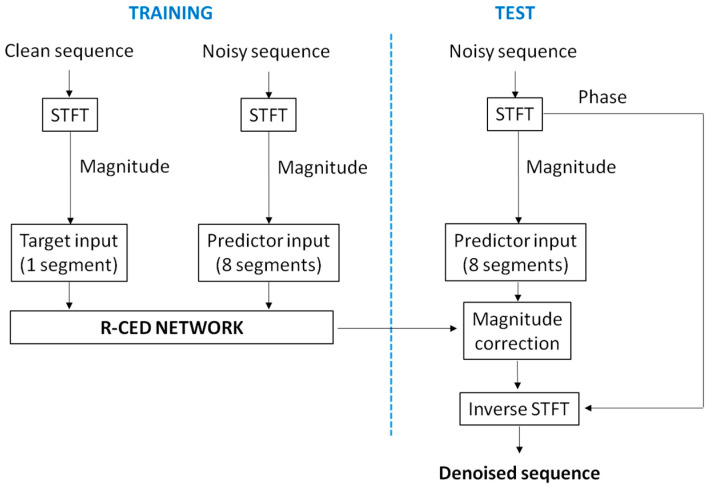
Flow chart of training and test stages for the proposed denoising algorithm.

The trained network was tested only on noisy sequences from the test set. Again, the test predictors were comprised of 8 consecutive STFT magnitude spectra from noisy sequences and normalized by the mean and standard deviation computed during the training stage. The trained network was applied to normalized test predictors, when denoised magnitude STFT were obtained. These magnitude spectra of the denoised sequences were then converted back to the time domain by applying the phase of the noisy sequences and the inverse STFT [34]. This way, as the final output, we obtained the denoised sequences of noisy test sequences. The flow chart of training and test stages is presented in Figure 3.

In order to measure the performance of the denoising algorithm, we applied the Pan and Tompkins R-peak detection algorithm on denoised and their corresponding clean and noisy armband sequences [35]. We chose the Pan and Tompkins’ algorithm, because it is still considered as one of the gold-standard ECG peak detection algorithms, and many algorithms are benchmarked against it. Detected peaks were visually inspected by three independent experts. The first and the last second of each sequence were not considered because of edge effects, and all analyses were performed on 8-s segments. In addition, we calculated several signal quality indices (SQI): signal-to-noise ratio (SNR), ratio of power [36], and cross-correlation with respect to clean sequences. SNR is defined as the ratio of power of the signal to the power of noise and is expected to be higher for clean signals than for noise-corrupted signals. The overall SNR is evaluated by calculating the improvement, *SNR_imp_*, using:

(1) $$SNR_{imp}=\;SNR_{denoised}-\;SNR_{noisy}$$

where *SNR_denoised_* is SNR obtained from denoised sequences, while *SNR_noisy_* is from noisy sequences [10]. Ratio of power is defined as the ratio of power in the frequency range of 5 to 20 Hz to the total power and is also expected to have a higher value when it is computed from a clean signal than from a noisy one. Cross-correlation with respect to clean sequences measures the similarity of a sequence of interest to its corresponding clean sequence and is expected to have a higher value and be closer to 1 for denoised sequences and be smaller for noisy sequences.

## 3. Results

### 3.1. Armband Sequences with Added Colored Noises

The trained R-CED network was tested on six 10-s sequences corrupted with added noise (white, blue, pink, purple, and brown) of two different levels for each color (total of 60 test sequences). Results of the Pan and Tompkins R-peak detection algorithm are presented in Table 1. The percent of correctly detected peaks in denoised sequences is higher than it is for sequences with all types of noise sources examined.

The improvement in SNR values, *SNR_imp_*, after applying the proposed denoising algorithm on noisy sequences is shown in Table 2. All the values are presented as mean ± standard deviation (sd). The algorithm achieved significant improvement in the quality of sequences when any of the five noise sources with both levels was added to clean sequences, which shows that the proposed denoising technique is effective.

The ratios of power for clean (reference), noisy, and denoised sequences are presented as mean ± sd in Table 3. We performed repeated measures ANOVA with the Bonferroni post-hoc test, with *p* < 0.05 considered as statistically significant, and obtained statistically significant differences between mean values of ratio of power of noisy and clean sequences as well as between noisy and denoised sequences, for all five types of noise and noise levels for each color.

The cross-correlation values between noisy and clean sequences, as well as between denoised and clean sequences, are shown in Table 4 as mean ± sd. The independent t-test showed that cross-correlation values between noisy and clean sequences were statistically significantly lower than cross-correlation values between denoised and clean sequences for all types of noise and all levels.

### 3.2. Armband Sequences with Motion Noise Artifacts

We tested the trained redundant convolutional encoder–decoder network on 28 noisy sequences. The results of R-peak detection are shown in Table 5. As can be noted from Table 5, the percent of correctly detected R-peaks for denoised sequences was the highest, 91.86%, compared to noisy sequences (61.16%) and clean (88.6%). Consequently, the percent of false positives for denoised sequences was the lowest, 8.14%. The possible explanation for not having the 100% peak detection accuracy for the clean sequences could be that the Pan and Tompkins algorithm is unable to detect all actual peaks for these sequences, probably because muscle movements are inevitable sometimes, and even for the very short duration, the signal that we consider as clean could have some short lasting artifacts.

Regarding SNR values, 19 out of 28 denoised sequences (67.86%) had higher SNR than the corresponding noisy ones. The overall sum of SNR values for denoised sequences was 96.83 dB, while the sum of SNR values for noisy sequences was lower, 85.91 dB. Moreover, for every test sequence, we calculated the improvement in SNR values using Equation (1) and obtained 0.39 ± 0.83 dB as mean ± sd. Note that with the proposed denoising technique, we achieved an overall increase in SNR values. Ratios of power for clean, noisy, and denoised sequences, represented as mean ± sd, were 0.74 ± 0.02, 0.61 ± 0.07 and 0.71 ± 0.03, respectively. Repeated measures ANOVA with the Bonferroni post-hoc test showed statistically significant differences between mean values of ratio of power of noisy and clean sequences, as well as between noisy and denoised sequences. Finally, cross-correlation between noisy and clean sequences was 0.74 ± 0.08 and was lower than between denoised and clean sequences 0.77 ± 0.06.

**Figure 4:**
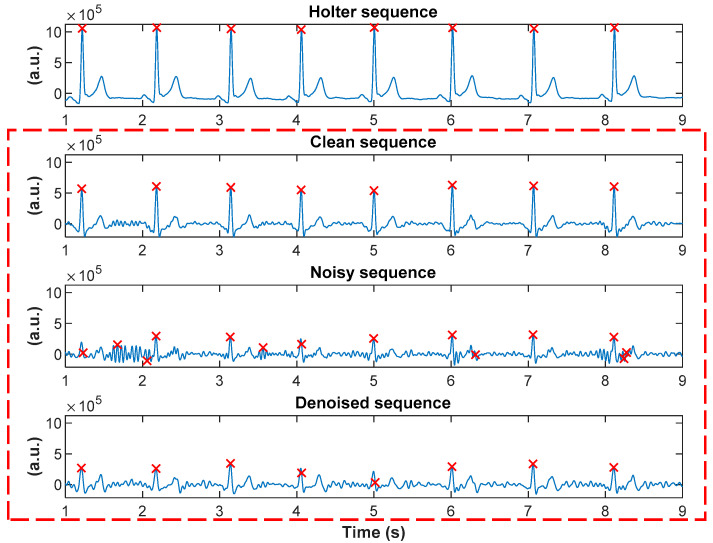
An example of the Holter sequence, corresponding clean armband sequence, a typical noisy armband sequence, and denoised signal after applying the proposed algorithm. R-peaks detected with the Pan and Tompkins algorithm are presented as red ‘x’ signs.

An illustrative example is shown in Figure 4. We present a sequence of Holter data, a time series from the clean armband channel for the same time range, the corresponding typical noisy armband channel, and denoised signal after applying our algorithm, as well as R-peaks detected (shown as red cross-marks) with the Pan and Tompkins algorithm. By comparing clean sequence with Holter, we can confirm that the recording from armband corresponds to the latter sequence, R-peaks coincide, and that it has the ECG morphology. As can be noted, the R-peak detection algorithm detected, wrongly, some additional peaks in the noisy sequence only. For this particular example, three independent experts visually inspected the sequence and determined that eight peaks were correctly detected by the algorithm in all four sequences, while for only the noisy sequence the algorithm detected six additional (wrong) peaks. The SNR of the noisy sequence was 2.27 dB, while the SNR of the denoised one was higher, 4.01 dB. Ratios of power for clean, noisy, and denoised sequences were 0.76, 0.51, and 0.70, respectively, while the cross-correlations of noisy and denoised sequences with respect to clean were 0.70 and 0.80, respectively. As can be noted, the signal quality is improved in the denoised sequence.

We compared the performance of the proposed R-CED algorithm with five other existing methods on sequences with motion noise artifacts recorded with the armband, namely algorithms based on discrete wavelet transformation (DWT) [14], empirical mode decomposition followed by discrete wavelet transform (EMD-DWT) [20], empirical mode decomposition adaptive switching mean filtering (EMD-ASMF) [22], sparsity-assisted signal smoothing (SASS) [9], and variable-frequency complex demodulation (VFCDM) [37]. The same evaluation measures were applied to all denoising methods: cross-correlation between denoised and clean sequences, ratio of power for denoised sequences, and the percent of correctly detected R-peaks in denoised sequences using Pan and Tompkins detection algorithm [35]. It can be seen from Table 6 that the proposed denoising algorithm provides the highest ratio of power for denoised sequences jointly with the VFCDM-based method. The cross-correlation between denoised and clean sequences of the proposed algorithm is significantly higher than that of the DWT-based method and very similar to those of the EMD-DWT-, EMD-ASMF-, SASS-, and VFCDM-based methods. The percent of correctly detected R-peaks for denoised sequences of the proposed algorithm is significantly higher than of the DWT-, EMD-DWT-, and SASS-based methods, while very similar to those of the EMD-ASMF- and VFCDM-based methods. It should be noted here that in the EMD-DWT-based method, QRS complexes are detected from the first three modes obtained using empirical mode decomposition, followed by soft thresholding-based wavelet denoising [20]. This method requires an external QRS complex detection for denoising of the ECG signal. Moreover, possible QRS complex candidates are detected from the first three modes of EMD, which may contain high-frequency spike-like noises and may result in the wrong QRS complex detection and, therefore, affect the performance of the denoising algorithm. Similarly, the EMD-ASMF-based method applies wavelet denoising on the first three EMD modes, which are then added with remaining modes in order to reconstruct a noise-free ECG sequence [22]. Subsequently, several postprocessing steps were performed, including application of ASMF. Since the ASMF significantly attenuates the QRS complex, this method requires an additional peak correction step. Therefore, similar to EMD-DWT, EMD-ASMF requires an external QRS complex detection before applying ASMF in order to correct the QRS complex peaks afterwards. Moreover, normal EMD has a mode-mixing problem [38], which may affect the denoising performance as well. The SASS problem formulation is expressed in terms of banded Toeplitz matrices and, thus, with fast solvers for banded systems of linear equations [9]. SASS-based method can be a computationally efficient denoising method; however, the overall performance of the SASS denoising was found to be suboptimal. In the VFCDM-based method, possible QRS-complex candidates are generated in the process of denoising in order to use only the QRS complex contribution from VFCDM modes 2–4, which is then added to the first VFCDM mode in order to reconstruct the noise-free sequence [37]. Therefore, if there are any significantly higher amplitude noise peaks in the ECG, they may be detected as potential QRS peak candidates and could be retained in the final denoised sequence. On the contrary, the proposed R-CED denoising algorithm does not use any postprocessing steps and, therefore, is more of a general algorithm than the five other compared methods. In addition, once trained, the R-CED algorithm can be much faster compared to EMD-DWT-based and EMD-ASMF-based methods, since latter require an external QRS complex detection and other postprocessing steps.

### 3.3. Sequences from MIT-BIH Arrhythmia Database

We performed two tests on recordings from the MIT-BIH arrhythmia database: (1) the denoising algorithm trained on 10-s armband sequences was applied to ten 60-s test sequences from the MIT-BIH database corrupted with added white noise of −5 dB SNR (denoted as test 1 in the text), and (2) the denoising algorithm trained on eleven 60-s sequences from the MIT-BIH database and their corresponding sequences corrupted with added white noise of −5 dB SNR was tested on ten 60-s sequences from the MIT-BIH database also corrupted with added white noise of −5 dB SNR (denoted as test 2 in the text). The SNR improvements for both denoising tests are shown in Table 7.

**Figure 5:**
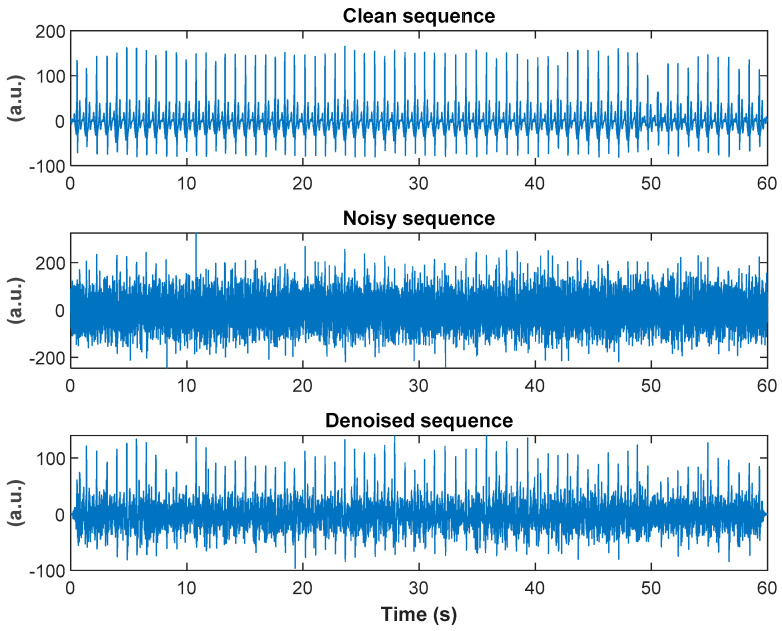
Illustrative example of one typical 60-s sequence from the MIT-BIH arrhythmia database and its corresponding noisy (corrupted with white noise with SNR = −5 dB) and denoised sequences.

In both cases, the denoising algorithm achieved improvements in SNR values of more than 7 dB. Note that in test 1 the algorithm was trained solely on sequences acquired by our wearable armband device and achieved very small standard deviation (0.25 dB). It is worth emphasizing again that the duration of training sequences was only 10 s. An example of one typical 60-s sequence and its corresponding noisy (corrupted with white noise with SNR = −5 dB) and denoised sequences is shown in Figure 5.

## 4. Discussion

An algorithm for denoising of electrocardiogram signals is presented. Long-term ECG signals were recorded with a wearable armband device that was worn by one male participant for 24 h during daily life routines on the upper left arm. Three different datasets were used for testing the proposed denoising algorithm: (1) recordings from armband device with added colored noises; (2) armband recordings corrupted with motion artifacts; (3) recordings from the MIT-BIH arrhythmia database [28,29].

The R-CED network was used as a denoising algorithm. The trained R-CED network was tested on noisy test sequences, and the performance of the proposed algorithm was measured by applying the Pan and Tompkins R-peak detection algorithm and by calculating signal quality indices (SNR improvement, ratio of power, and cross-correlation with respect to the clean sequences).

When the denoising algorithm was applied to sequences corrupted with added noise, the percentage of correctly detected R-peaks in denoised sequences was higher than in noisy sequences—we achieved 100% accuracy in denoised sequences when blue and purple noises were added. The improvement in SNR values varied between 7 dB and 19 dB. The ratios of power for noisy sequences were significantly lower when compared to those of both clean and denoised sequences. Similarly, cross-correlations between noisy and clean sequences were significantly lower than between denoised and clean sequences.

The results on the second dataset showed that the R-peaks in denoised sequences were detected with the highest accuracy, 91.86%, followed by clean (88.6%) and noisy sequences (61.16%). The improvement in SNR values was 0.39 ± 0.83 (mean ± sd). Ratios of power for clean, noisy, and denoised sequences were 0.74 ± 0.02, 0.61 ± 0.07, and 0.71 ± 0.03, respectively, with statistically significant differences between noisy and clean sequences, as well as between noisy and denoised sequences. The cross-correlation between noisy and clean sequences was 0.74 ± 0.08 and was lower than between denoised and clean sequences (0.77 ± 0.06). As can be observed, the proposed denoising algorithm significantly improved the quality of the signal. Note that, since the signals were recorded during daily life routines, noisy signals were corrupted with motion artifacts and muscle-induced noises.

These results were compared to results obtained by applying five other methods on the same dataset. We applied methods based on: DWT [14], EMD-DWT [20], EMD-ASMF [22], SASS [9], and VFCDM [37]. The proposed denoising algorithm, R-CED network, achieved the highest ratio of power for denoised sequences jointly with the method based on VFCDM. Regarding the cross-correlation between denoised and clean sequences, the proposed algorithm obtained a significantly higher value than DWT-based method and a very similar value to EMD-DWT-, EMD-ASMF-, SASS-, and VFCDM-based methods. The percent of correctly detected R-peaks for denoised sequences of the proposed algorithm achieved a significantly higher value than DWT-, EMD-DWT-, and SASS-based methods and a very similar value to EMD-ASMF- and VFCDM-based methods. It is worth mentioning that both EMD-DWT- and EMD-ASMF-based methods require an external QRS complex detector and additional postprocessing steps in order to correct the R-peaks. Similarly, VFCDM-based method also requires additional postprocessing steps during the denoising process. In contrast, the proposed R-CED denoising algorithm does not use any postprocessing steps, which makes this algorithm a more general method. In addition, once trained, R-CED algorithm performs much faster than the other five existing methods. These are the few advantages and innovations of the proposed algorithm.

Two tests were performed on sequences from the MIT-BIH arrhythmia database. When the denoising algorithm, trained on 10-s armband sequences was tested on 60-s sequences from the arrhythmia database, the improvement in SNR value was 7.08 ± 0.25 dB (mean ± sd). Moreover, when the denoising algorithm was trained and tested on 60-s sequences from the same database, we achieved 7.43 ± 0.81 dB (mean ± sd) SNR improvement.

Other denoising techniques have been reported in the literature. Sayadi and Shamsollahi presented an ECG denoising algorithm based on the modified extended Kalman filter structure and validated the performance on recordings from the MIT-BIH arrhythmia database [12]. The authors showed improvements in SNR values. We used the same test sequences from the same time frames as reported in [12]. The SNR improvement after applying our algorithm is slightly lower (~2 dB in mean value) than in [12], but we achieve notably lower standard deviation (0.25 dB as opposed to 0.52 dB). It is worth noting that our algorithm has several advantages over the algorithm proposed in [12]. The first advantage is that the standard deviation achieved is twice as low and the mean value of SNR improvement is only slightly lower. Note that our results were obtained when the denoising algorithm was trained with sequences that are not from the MIT-BIH database but from sequences recorded with our wearable device. The second advantage is that the algorithm based on Kalman filters is adaptive and needs time to adjust and track the signal at hand, whereas our algorithm does not need that additional time to adapt to the signal. Lastly, our denoising algorithm is trained once and can be applied to any sequence of any duration, as opposed to the algorithm proposed in [12] that needs to be adjusted every time a new sequence is used for testing and cannot be used on sequences of short durations (e.g., 10-s sequences).

Akhbari et al. applied a denoising algorithm on MIT-BIH arrhythmia and noise stress test databases and achieved good SNR improvement [23]. Even though we used the same test recordings as in [23], a direct comparison of results is not possible because the authors did not provide the time frames within recordings used for their analysis. Xiong et al. proposed a stacked contractive denoising autoencoder to denoise ECG sequences [25] and applied it to ECG signals from the MIT-BIH arrhythmia database and noise from the MIT-BIH noise stress test database. The results reported in [25] showed improvements in the signals; however, they were reported in a sequence-based manner and not overall, and the time frames of sequences used were not stated in the paper, thus we cannot make direct comparison to our results. Zhou et al. applied denoising algorithm to sequences from the MIMIC II database and to sequences with added simulated noise and performed QRS complex detection [24]. The direct comparison between our results and results from [24] cannot be performed, due to the differences in reported performance measures. B’charri et al. proposed an algorithm based on the dual tree wavelet transform and used simulated ECG recordings as well as the MIT-BIH arrhythmia and noise stress test databases [17]. Again, the direct comparison of our results with results presented in [17] cannot be performed due to the fact that the authors of [17] presented results in a sequence-based and not overall manner, and the time frames of sequences used was not stated in the paper. Hesar and Mohebbi utilized the marginalized particle extended Kalman filter as a denoising algorithm and evaluated it on the MIT-BIH normal sinus rhythm and arrhythmia databases, where sequences in both databases were contaminated with Gaussian white noise and noise from the MIT-BIH noise stress test database with predefined noise levels [13]. The direct comparison between our results and results from [13] cannot be performed due to the differences in reported performance measures.

Most importantly, as shown in Table 5, the denoised signal using our approach resulted in 91.86% (from 61.16% for the noise contaminated), whereas for the clean signal, it was only 88.6% accurate in correctly detecting QRS complexes. This suggests our method improves the signal quality of even the clean signal and surpasses the performance of one of the most cited peak detection methods [35]. Therefore, this result suggests the proposed algorithm is quite effective in filtering out undesired noise sources, which preclude accurate QRS complex detection. Moreover, the proposed algorithm does not require any postprocessing steps nor any ECG signals from external devices for high denoising performance, which makes this algorithm a more general and faster method than other existing denoising methods.

## 5. Conclusions

In this study, we proposed a deep learning algorithm—a redundant convolutional encoder–decoder network—for denoising electrocardiogram signals recorded with a wearable armband device for 24 h. The results suggest that the proposed denoising algorithm significantly improved the quality of the ECG signals corrupted with additive noise of various types and motion artifact noise. We obtained high accuracy of correctly detected R-peaks in denoised sequences, high values of SNR improvement in denoised sequences, significantly higher ratios of power for denoised sequences than for noisy ones, and significantly higher cross-correlation values between denoised and clean sequences than between noisy and clean ones. Note that the main goal of this study was to obtain reliable R-peaks so that accurate heart-rate estimates could be obtained, which makes this algorithm an appealing tool for addressing cardiac-related issues, such as cardiac arrhythmia detection. It is worth mentioning that the proposed denoising algorithm does not require any postprocessing steps, nor any additional/external ECG signals—it solely uses ECG signals recorded with the same wearable armband device, which is the main advantage and innovation of the proposed algorithm. In addition, the proposed algorithm can be applied to sequences of any duration and signals recorded with various ECG devices to achieve good results. Further studies will be conducted on additional recordings to obtain a generalized trained network that can be applied to larger databases and to further improve denoising results. In addition, in the future study, we will perform an investigation on the complexities of embedding our algorithm into an ECG measurement device.

## Figures and Tables

**Table 1:** The percent of correctly detected peaks in clean (reference), noisy, and denoised sequences.

	Clean (Reference)	Noisy	Denoised
White noise, SNR −5 dB	100%	80%	95.92%
White noise, SNR −7 dB	100%	83.87%	86.54%
Blue noise, SNR −5 dB	100%	97.78%	100%
Blue noise, SNR −7 dB	100%	95.65%	100%
Pink noise, SNR −5 dB	100%	62.5%	93.75%
Pink noise, SNR −7 dB	100%	66.67%	70.49%
Purple noise, SNR −5 dB	100%	93.62%	100%
Purple noise, SNR −7 dB	100%	95.65%	100%
Brown noise, SNR −15 dB	100%	43.56%	80.85%
Brown noise, SNR −17 dB	100%	34.33%	79.17%

**Table 2:** *SNR_imp_* expressed as mean ± sd.

	*SNR_imp_* (dB) Mean ± sd
White noise, SNR −5 dB	7.32 ± 0.2
White noise, SNR −7 dB	8.43 ± 0.17
Blue noise, SNR −5 dB	8.76 ± 0.24
Blue noise, SNR −7 dB	9.9 ± 0.49
Pink noise, SNR −5 dB	6.45 ± 0.26
Pink noise, SNR −7 dB	7.64 ± 0.42
Purple noise, SNR −5 dB	8.81 ± 0.26
Purple noise, SNR −7 dB	9.77 ± 0.33
Brown noise, SNR −15 dB	17.39 ± 1.11
Brown noise, SNR −17 dB	18.79 ± 1.73

**Table 3:** Ratio of power for clean, noisy, and denoised sequences and the *p*-values.

	Clean (Reference)	Noisy (*p*-Values)	Denoised (*p*-Values)
White noise, SNR −5 dB	0.73 ± 0.02	0.26 ± 0.01 *^&^ (<3 × 10^−8^)	0.63 ± 0.01 * (<2 × 10^−5^)
White noise, SNR −7 dB		0.22 ± 0.01 *^&^ (<2 × 10^−7^)	0.61 ± 0.01 * (<6 × 10^−5^)
Blue noise, SNR −5 dB		0.20 ± 0.01 *^&^ (<2 × 10^−8^)	0.60 ± 0.02 * (<2 × 10^−5^)
Blue noise, SNR −7 dB		0.15 ± 0.01 *^&^ (<5 × 10^−6^)	0.55 ± 0.04 * (<2 × 10^−4^)
Pink noise, SNR −5 dB		0.32 ± 0.02 *^&^ (<7 × 10^−6^)	0.64 ± 0.02 * (<2 × 10^−4^)
Pink noise, SNR −7 dB		0.28 ± 0.02 *^&^ (<1 × 10^−6^)	0.62 ± 0.01 * (<2 × 10^−4^)
Purple noise, SNR −5 dB		0.17 ± 0.00 *^&^ (<3 × 10^−7^)	0.53 ± 0.02 * (<2 × 10^−6^)
Purple noise, SNR −7 dB		0.13 ± 0.01 *^&^ (<3 × 10^−6^)	0.47 ± 0.03 * (<2 × 10^−5^)
Brown noise, SNR −15 dB		0.03 ± 0.02 *^&^ (<4 × 10^−7^)	0.63 ± 0.02 * (<4 × 10^−5^)
Brown noise, SNR −17 dB		0.02 ± 0.01 *^&^ (<2 × 10^−7^)	0.59 ± 0.02 * (<2 × 10^−4^)

* presents statistically significant difference with respect to clean (reference) sequences; & presents statistically significant difference with respect to denoised sequences.

**Table 4:** Cross-correlation results with respect to clean (reference) sequences represented as mean ± sd, and the *p*-values.

	Noisy Sequences (*p*-Values)	Denoised Sequences
White noise, SNR −5 dB	0.49 ± 0.01 * (4 × 10^−12^)	0.71 ± 0.01
White noise, SNR −7 dB	0.41 ± 0.01 * (4 × 10^−11^)	0.64 ± 0.01
Blue noise, SNR −5 dB	0.48 ± 0.01 * (9 × 10^−13^)	0.79 ± 0.01
Blue noise, SNR −7 dB	0.41 ± 0.01 * (1 × 10^−10^)	0.74 ± 0.03
Pink noise, SNR −5 dB	0.50 ± 0.02 * (1 × 10^−7^)	0.64 ± 0.02
Pink noise, SNR −7 dB	0.43 ± 0.03 * (6 × 10^−5^)	0.57 ± 0.04
Purple noise, SNR −5 dB	0.48 ± 0.01 * (2 × 10^−13^)	0.79 ± 0.01
Purple noise, SNR −7 dB	0.41 ± 0.01 * (8 × 10^−12^)	0.74 ± 0.02
Brown noise, SNR −15 dB	0.23 ± 0.04 * (2 × 10^−7^)	0.71 ± 0.08
Brown noise, SNR −17 dB	0.27 ± 0.18 * (0.002)	0.65 ± 0.13

* presents statistically significant difference to denoised sequences.

**Table 5:** Peak detection results on clean (reference), noisy, and denoised sequences.

	Clean (Reference)	Noisy	Denoised
Correctly detected peaks	88.60%	61.16%	91.86%
Missed peaks	11.40%	38.84%	8.14%

**Table 6:** Denoising performance comparison on the armband sequences with motion noise artifacts: cross-correlation between denoised and clean sequences represented as mean ± sd, ratio of power for denoised sequences represented as mean ± sd, and percent of correctly detected peaks in denoised sequences.

Method	Cross-Correlation	Ratio of Power	Correctly Detected Peaks
DWT-based [14]	0.74 ± 0.08	0.63 ± 0.06	76.28%
EMD-DWT-based [20]	0.77 ± 0.07	0.70 ± 0.04	87.27%
EMD-ASMF-based [22]	0.79 ± 0.06	0.70 ± 0.03	92.83%
SASS-based [9]	0.79 ± 0.07	0.69 ± 0.05	84.98%
VFCDM-based [37]	0.81 ± 0.06	0.71 ± 0.03	92.83%
Proposed method	0.77 ± 0.06	0.71 ± 0.03	91.86%

**Table 7:** SNR improvements for denoising algorithm trained on armband sequences (test 1) and sequences from MIT-BIH arrhythmia database (test 2).

	Test 1	Test 2
Mean ± sd (dB)	7.08 ± 0.25	7.43 ± 0.81
Max (dB)	7.55	9.03
Min (dB)	6.78	6.32
